# Supplementary material for: Conformal Invariance of Graphene Sheets
Source: Sci Rep. 2016 Mar 10;6:22949. doi: 10.1038/srep22949 (PMC4785365; doi:10.1038/srep22949)
Supplement: Supplementary Information [file srep22949-s1.pdf]

Supplementary Material: Conformal Invariance of Graphene Sheets

I.Giordanelli[1,*], N. Posé[1], M. Mendoza[1], and H. J. Herrmann[1,2]

[1]ETH Zürich, Computational Physics for Engineering Materials, Institute for Building Materials, Wolfgang-Pauli-Strasse 27, HIT, CH-8093 Zürich, Switzerland
[2]Universidade Federal do Ceará, Departamento de Física, Campus do Pici, 60455-760 Fortaleza, Ceará, Brazil
*gilario@ethz.ch

ABSTRACT

This supplementary material contains some additional details regarding the velocity distribution in the molecular dynamics simulation, the power spectrum of the Fourier transform of the graphene samples, further details concerning the fractal dimension, the Left-passage probability test within the SLE theory, and finally some remarks on other atomistic membranes.

Maxwell-Boltzmann distribution

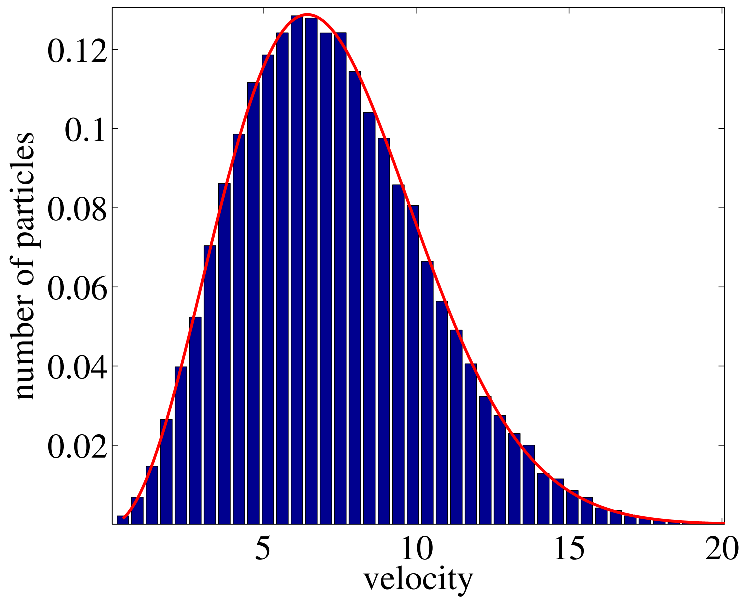

Figure S1. Velocity distribution of carbon atoms of a quadratic graphene sheet of size 400 Å after 200 ps simulation. The red line is the Maxwell-Boltzmann distribution at $T = 300$ K.

Power spectrum

We study some further properties of the graphene sheets, and especially its power spectrum. From the power spectrum of a graphene membrane of size 800 Å at 300 K, we obtain an estimate of the Hurst exponent α (see Fig. S2) in agreement with the one obtained from the height-height correlation function, see Fig. 2 of the main text.

Fractal dimension at higher temperatures

We perform an additional study to see if the fractal dimension of the iso-height contour lines keeps being independent of the system size for higher temperatures. Indeed, Fig. S3 confirms the temperature independence of the system size for temperatures

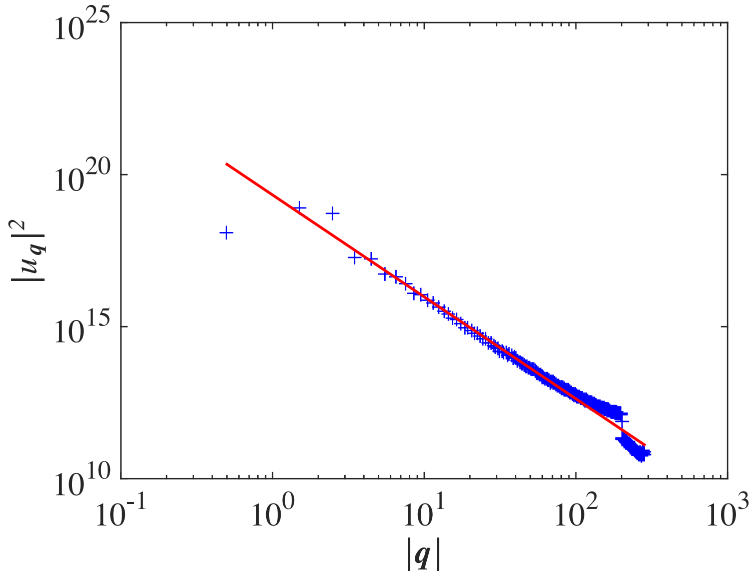

Figure S2. Power spectrum of a given graphene sheet. One recovers the expected power law behavior. The solid line is a guide to the eye of a slope of $-2(\alpha+1)$ with $\alpha = 0.68 \pm 0.05$.

much higher than 600 K.

Fractal dimension of the area

The fractal dimension d_a of the area that the contour lines enclose has also been measured (see Fig. S4), finding a value of $d_a = 1.82 \pm 0.01$, which is also independent of the temperature. Our results show that the iso-height contour lines and the area enclosed by them possess scale invariant properties. We have also studied the fractal dimension of the watershed (defined in Ref.[1]) of each graphene sample. A temperature invariant fractal dimension of those lines is found to be $d_f = 1.07 \pm 0.01$. This result strengthens the analogy of graphene membranes with other landscapes at criticality.[2]

Left-passage probability

In order to have a further numerical evidence of the compatibility of the statistics of iso-height lines on graphene surfaces with SLE, we study a further property of the SLE paths. Their left-passage probabilities should follow the so-called Schramm's formula of Eq. (1) for *chordal* SLE curves. Such curves start at the origin and grow towards infinity. Therefore, they split the upper half-plane in two domains: the points that are at the left of the curve, and the ones that are at the right. Schramm provided an expression for the probability that the curve goes at the left of a given point in the upper half-plane, i.e. the point is on the right side of the curve:

$$P_\kappa(\phi) = \frac{1}{2} + \frac{\Gamma(4/\kappa)}{\sqrt{\pi}\,\Gamma\left(\frac{8-\kappa}{2\kappa}\right)} \cot(\phi)\, {}_2F_1\left(\frac{1}{2}, \frac{4}{\kappa}, \frac{3}{2}, -\cot(\phi)^2\right), \tag{1}$$

where Γ is the Gamma function and ${}_2F_1$ is the hypergeometric function. Here we show that this formula is satisfied by the iso-height lines of graphene using the following method. We first define a set of sample points S, for which we estimate the probability of being at the right of our curves that have been mapped to infinity.[3] To estimate κ, we minimize the mean square deviation $Q(\kappa)$ defined as:

$$Q(\kappa) = \frac{1}{|S|} \sum_{z \in S} [P(z) - P_\kappa(\phi)]^2, \tag{2}$$

where $|S|$ is the cardinality of the set S, and $P(z)$ the measured left-passage probability at the point z. The value of κ for which the minimum of Q is attained gives us an estimate of the diffusion coefficient of our iso-height lines. In Fig. S5, we can see that the results are in agreement with our previous estimates of $\kappa = 2.27 \pm 0.08$, and also show that the left-passage probability satisfies Schramm's formula.

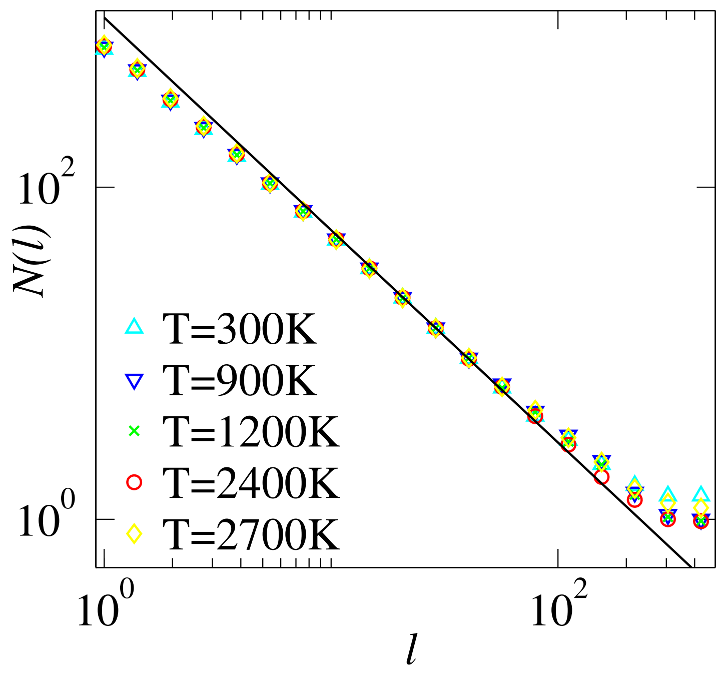

Figure S3. Fractal dimension of the iso-height contour lines computed with the yardstick method for different temperatures and fixed system size of 800 Å.

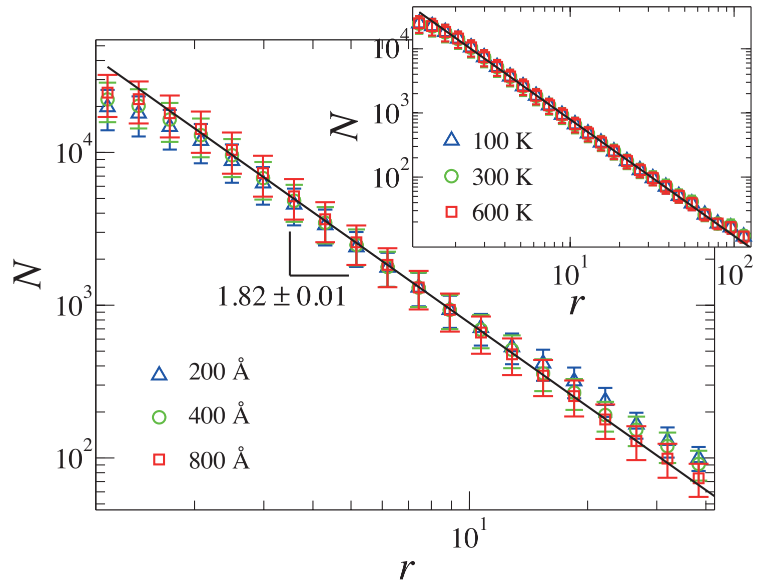

Figure S4. Fractal dimension of the area enclosed by the iso-height contour lines measured with the box-counting method. Main panel: The fractal dimension for different system sizes at $T = 300$ K. Inset: The fractal dimension for different temperatures for a system size of 800 Å.

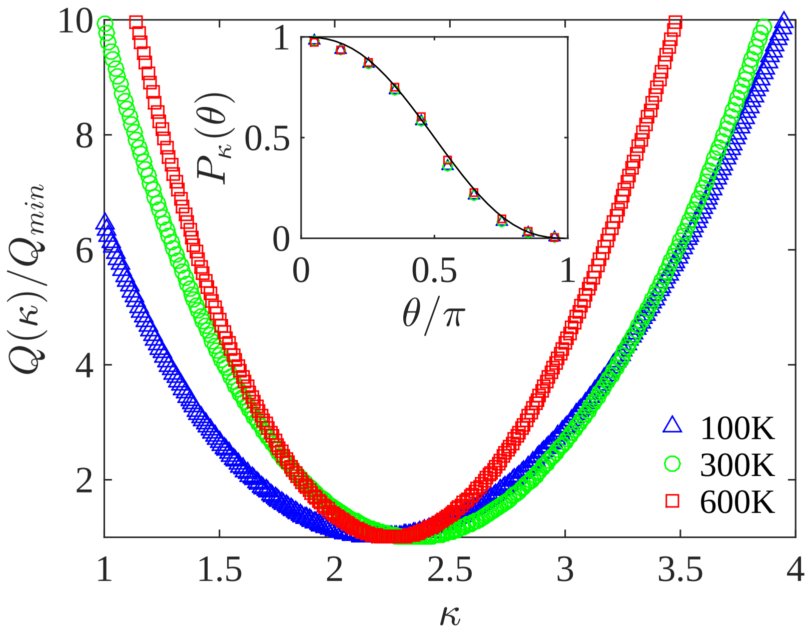

Figure S5. (color online) Measured rescaled mean square deviation $Q(\kappa)/Q_{min}$ as a function of κ being Q_{min} the minimum value of Q, for temperatures $T = 100$ K, $T = 300$ K, and $T = 600$ K. Inset: the measured left-passage probabilities are compared with Schramm's formula for $\kappa = 2.24$ (displayed as the solid line).

Other atomistic membranes

Fig. S6 shows a simulation performed with silicon atoms in a honeycomb lattice. We can see that the suspended structure crumples and does not form a stable two-dimensional-like crystal, in contrast to graphene membranes (see inset of Fig. S6). The stability of the suspended graphene membrane is not found for the chemically similar suspended silicene membrane.

References

1. Fehr, E. *et al.* New efficient methods for calculating watersheds. *Journal of Statistical Mechanics: Theory and Experiment* **2009**, P09007 (2009).
2. Fehr, E., Kadau, D., Araújo, N. A. M., Andrade Jr., J. S. & Herrmann, H. J. Scaling relations for watersheds. *Phys. Rev. E* **84**, 036116 (2011).
3. Posé, N., Schrenk, K. J., Araújo, N. A. M. & Herrmann, H. J. Shortest path and Schramm-Loewner Evolution. *Sci. Rep.* **4**, 5495 (2014).
4. Tersoff, J. New empirical approach for the structure and energy of covalent systems. *Phys. Rev. B* **37**, 6991–7000 (1988).

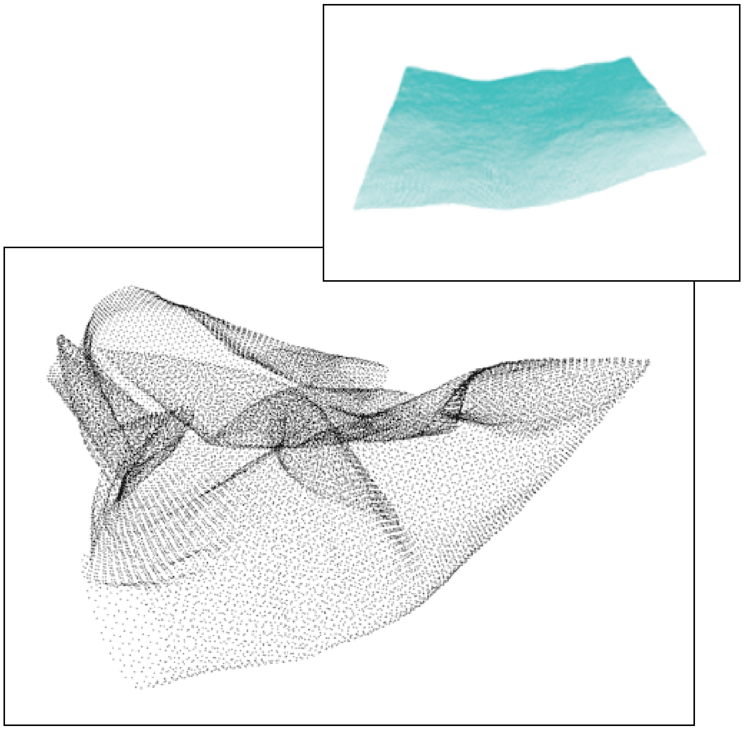

Figure S6. Main panel: Simulation of suspended silicene, a two-dimensional honeycomb lattice with silicon atoms. The simulation has been performed with the Tersoff potential applied to silicon.[4] Inset panel: Graphene membrane simulated with the Tersoff potential.
